# Multiorgan Toxicity Following Rifapentine and Isoniazid Therapy for Latent Tuberculosis Infection: A Case Report

**DOI:** 10.7759/cureus.114990

**Published:** 2026-08-22

**Authors:** Shayan Parvini Najafabadi, Karthik Ramesh, Betty Tu, Brad Fiske, Nathaniel Meyer

**Affiliations:** 1 Department of Medicine, Ronald Reagan University of California at Los Angeles (UCLA) Medical Center, Los Angeles, USA

**Keywords:** 3hp regimen, acute kidney injury, drug-induced hypersensitivity, drug-induced liver injury (dili), hemolytic anemia, isoniazid, latent tuberculosis infection, multiorgan toxicity, rifapentine, thrombocytopenia

## Abstract

The once-weekly combination of rifapentine and isoniazid (3HP regimen) is a preferred treatment for latent tuberculosis infection (LTBI). Although generally well tolerated, rare but severe systemic adverse reactions may occur. We report the case of a 57-year-old woman receiving once-weekly rifapentine, isoniazid, and pyridoxine for LTBI who developed recurrent chest pain, generalized weakness, nausea, and vomiting within a short time after taking each weekly dose.

The evaluation demonstrated multiorgan involvement, including acute liver injury, hyperbilirubinemia, hyperferritinemia, markedly elevated lactate dehydrogenase (LDH), undetectable haptoglobin, thrombocytopenia, and acute kidney injury. An extensive evaluation for infectious, cardiac, hematologic, and hepatic etiologies was unrevealing. The temporal relationship to medication administration, recurrence with re-exposure, and improvement following discontinuation of the rifapentine and isoniazid regimen supported a diagnosis of severe drug-induced hypersensitivity with hepatic, hematologic, renal, and systemic inflammatory involvement. The patient was managed with supportive care, resulting in progressive improvement of laboratory abnormalities, although renal recovery remained incomplete at discharge.

This case highlights a rare but clinically significant multiorgan adverse reaction associated with the 3HP regimen. Clinicians should maintain a high index of suspicion for drug-induced hypersensitivity in patients who develop recurrent systemic symptoms after dosing, as early recognition and prompt discontinuation of therapy may prevent significant morbidity.

## Introduction

An estimated one-fourth of the global population harbors latent tuberculosis infection (LTBI) [1]. It is estimated that approximately 5%-15% of individuals with LTBI will progress to active tuberculosis during their lifetime, making the identification and treatment of LTBI a critical public health priority [2]. Current guidelines preferentially recommend short-course rifamycin-based regimens for LTBI treatment, including three months of once-weekly isoniazid plus rifapentine (3HP), four months of daily rifampin, or three months of daily isoniazid plus rifampin [3]. The 3HP regimen is generally well tolerated, and most adverse effects develop within the first three to four doses, present with flu-like symptoms, and resolve within 24 hours [4-6].

While the 3HP regimen is well-tolerated, we report a rare instance of severe multiorgan toxicity, characterized by acute hepatic, hematologic, and renal injury that challenges the typical presentation of self-limited systemic drug reactions. Herein, we describe the case of a 57-year-old woman receiving weekly rifapentine and isoniazid therapy who developed recurrent systemic symptoms culminating in multiorgan dysfunction.

To our knowledge, this represents a rare and severe reaction to a widely used therapy. Moreover, initial laboratory normalcy and confounding imaging findings further obfuscated the diagnosis. The case underscores the importance of recognizing serious adverse reactions associated with rifapentine/isoniazid therapy, maintaining a broad differential diagnosis in patients presenting with recurrent systemic symptoms after dosing, and promptly discontinuing the offending agents to minimize morbidity.

## Case presentation

A 57-year-old woman with recently diagnosed latent tuberculosis infection (LTBI) receiving once-weekly rifapentine, isoniazid, and pyridoxine (3HP regimen) was admitted with recurrent chest pain. She had no significant past medical history, including no known chronic liver disease, kidney disease, hematologic disorders, or autoimmune disease. Her only reported medication intolerance was to morphine, which had previously caused respiratory depression. The patient had initiated the 3HP regimen approximately three weeks prior to hospitalization.

Initial presentation and workup

**Figure 1:**
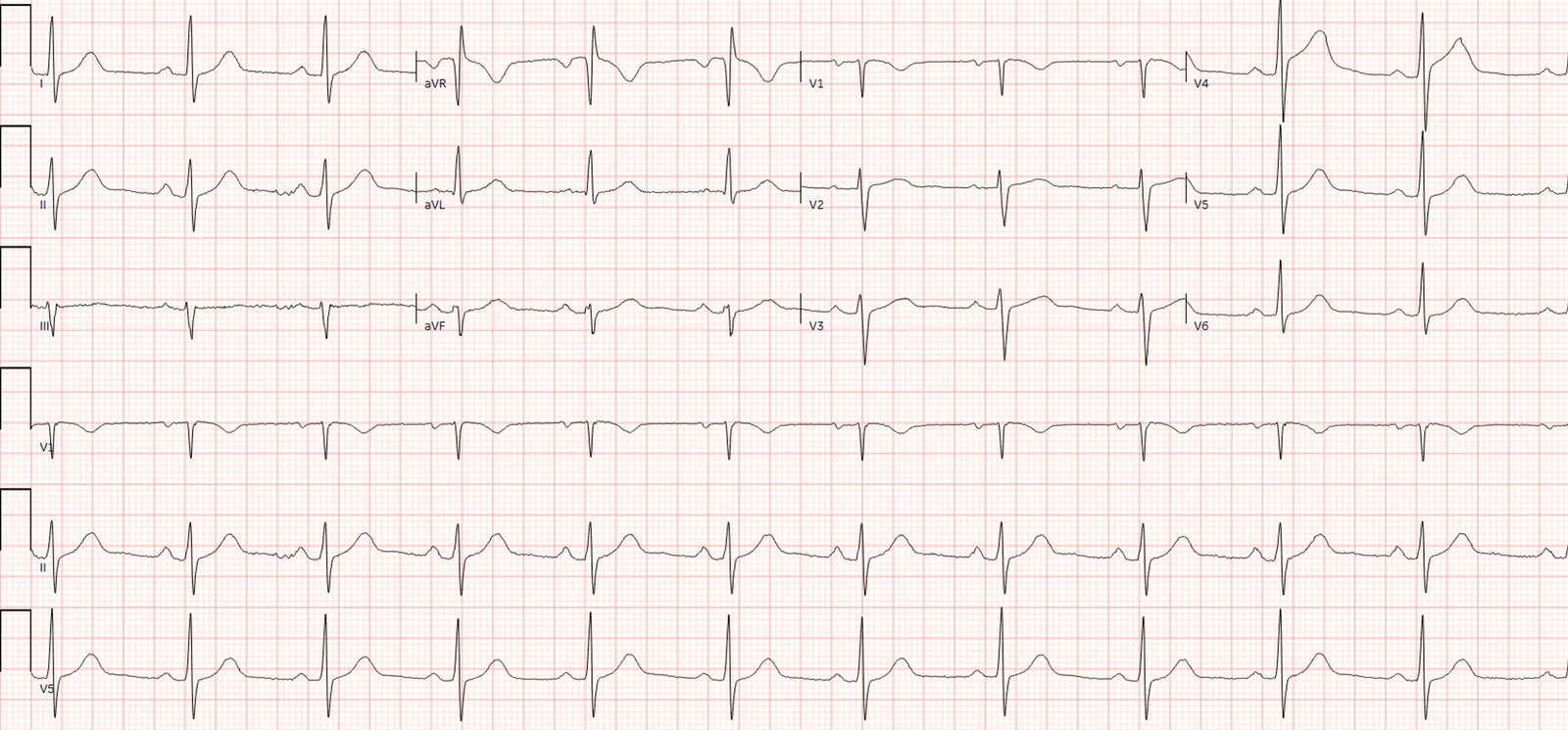
Initial 12-lead electrocardiogram obtained during the patient's first emergency department presentation, notable for normal sinus rhythm with left axis deviation.

**Figure 2:**
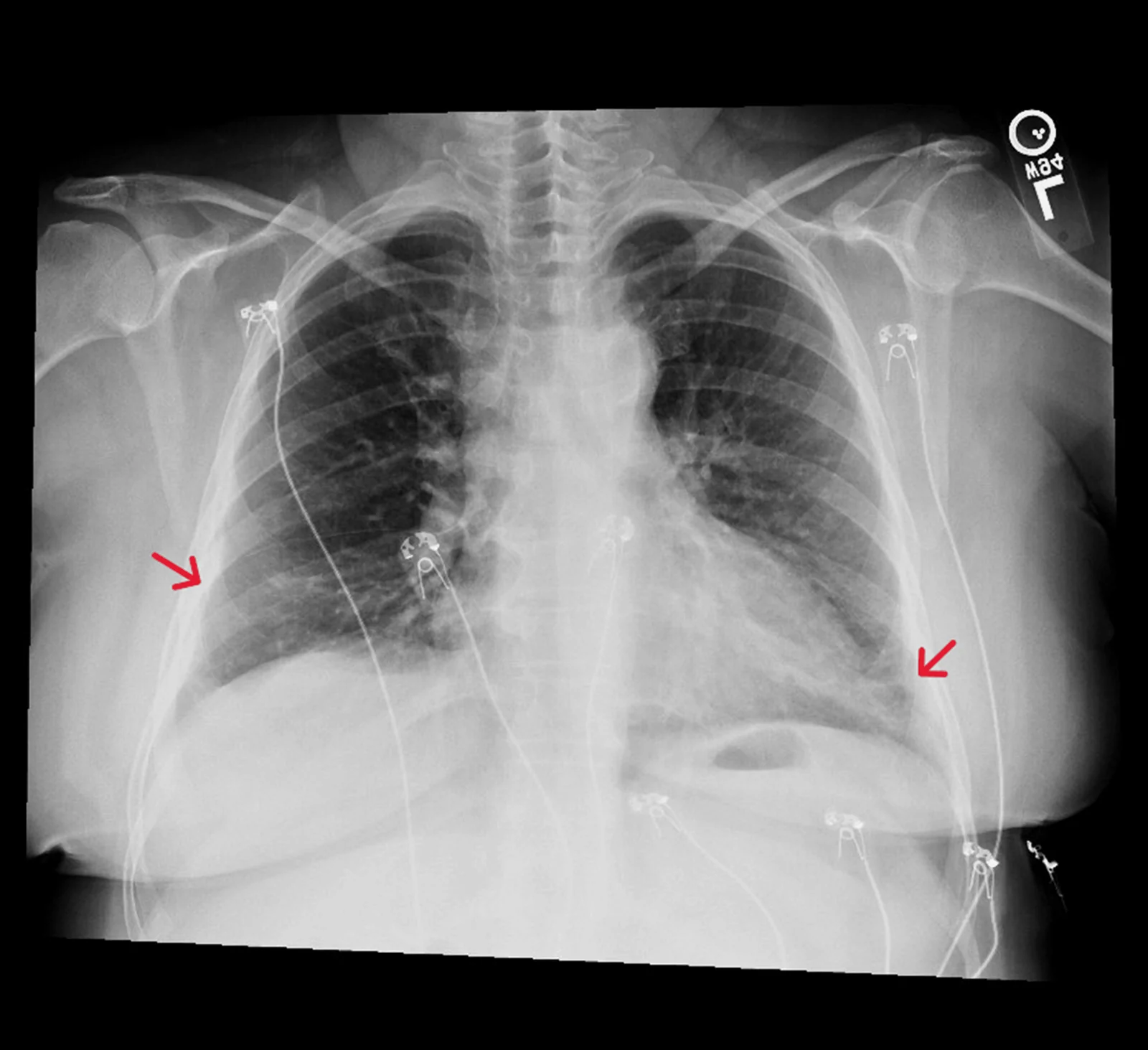
Initial chest radiograph obtained during the patient's first emergency department presentation demonstrating patchy bibasilar opacities. Anteroposterior chest radiograph. Red arrows indicate patchy bibasilar airspace opacities. These nonspecific findings were initially interpreted as concerning for atelectatic change versus early pneumonia.

The patient tolerated her first weekly dose of the 3HP regimen (isoniazid 900 mg and rifapentine 900 mg) without adverse effects. Approximately three hours after her second weekly dose, she developed diffuse myalgias, chills, vomiting, chest pain, and shortness of breath, prompting her first evaluation in the emergency department. Initial workup, including a complete blood count, basic metabolic panel, electrocardiogram (Figure 1), and respiratory viral panel, was unremarkable. Chest radiography demonstrated patchy bibasilar opacities (Figure 2). She was discharged home with a prescription for amoxicillin for presumed community-acquired pneumonia; however, she was not initiated on antibiotic therapy because her symptoms improved spontaneously after discharge. Over the following several days, her symptoms completely resolved, and she returned to her baseline state of health.

Second presentation and hospital course

Approximately three hours after taking her third weekly dose of rifapentine and isoniazid, the patient developed recurrent chest discomfort accompanied by generalized weakness, diffuse bone and muscle pain, nausea, and vomiting. She returned to the emergency department for further evaluation. On presentation, the patient appeared fatigued but was alert and oriented. Physical examination was notable for conjunctival icterus, hepatomegaly, and mild right upper quadrant tenderness to palpation.

Laboratory evaluation on admission demonstrated evidence of multiorgan involvement including acute liver injury (total bilirubin 11 mg/dL, conjugated bilirubin 4.3 mg/dL, aspartate aminotransferase (AST) 607 U/L, alanine aminotransferase (ALT) 192 U/L) and acute kidney injury (serum creatinine of 2.48 mg/dL and blood urea nitrogen of 43 mg/dL with baseline values of 0.66 mg/dL and 14 mg/dL, respectively). Notable hematologic abnormalities included anemia (hemoglobin 10.1 g/dL), thrombocytopenia (110×10⁹/L), markedly elevated lactate dehydrogenase (2,740 U/L), undetectable haptoglobin (<10 mg/dL), and hyperferritinemia (>20,000 ng/mL). The combination of anemia, markedly elevated lactate dehydrogenase (LDH), and undetectable haptoglobin was consistent with hemolysis. Direct antiglobulin testing was negative, peripheral blood smear demonstrated no schistocytes, and coagulation studies remained preserved throughout hospitalization. Despite a mild leukocytosis (white blood cell count, 14.26×10³/µL), the absolute eosinophil count remained within the normal range throughout hospitalization.

**Figure 3:**
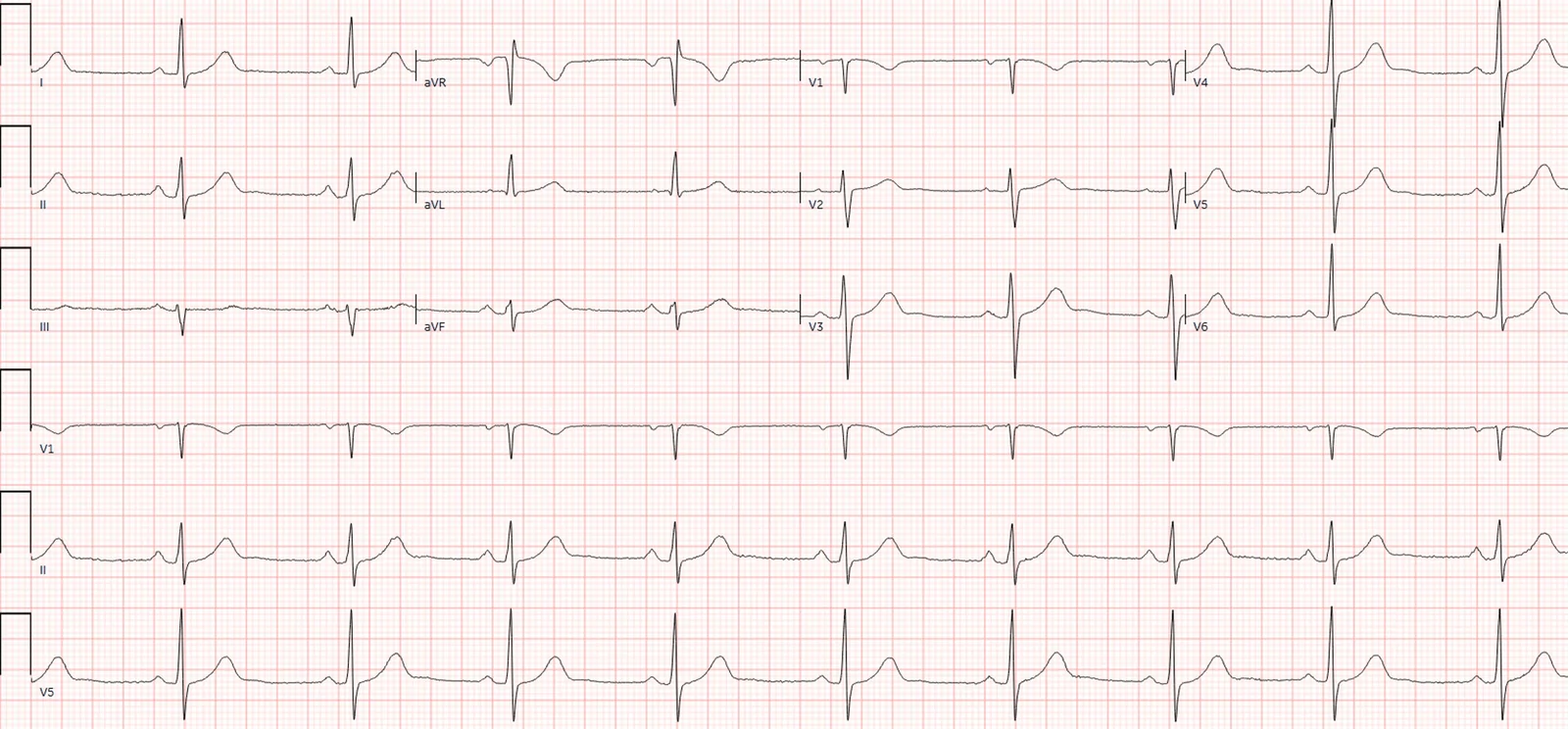
Twelve-lead electrocardiogram obtained during the patient's second emergency department presentation, demonstrating normal sinus rhythm with no acute ischemic changes.

**Figure 4:**
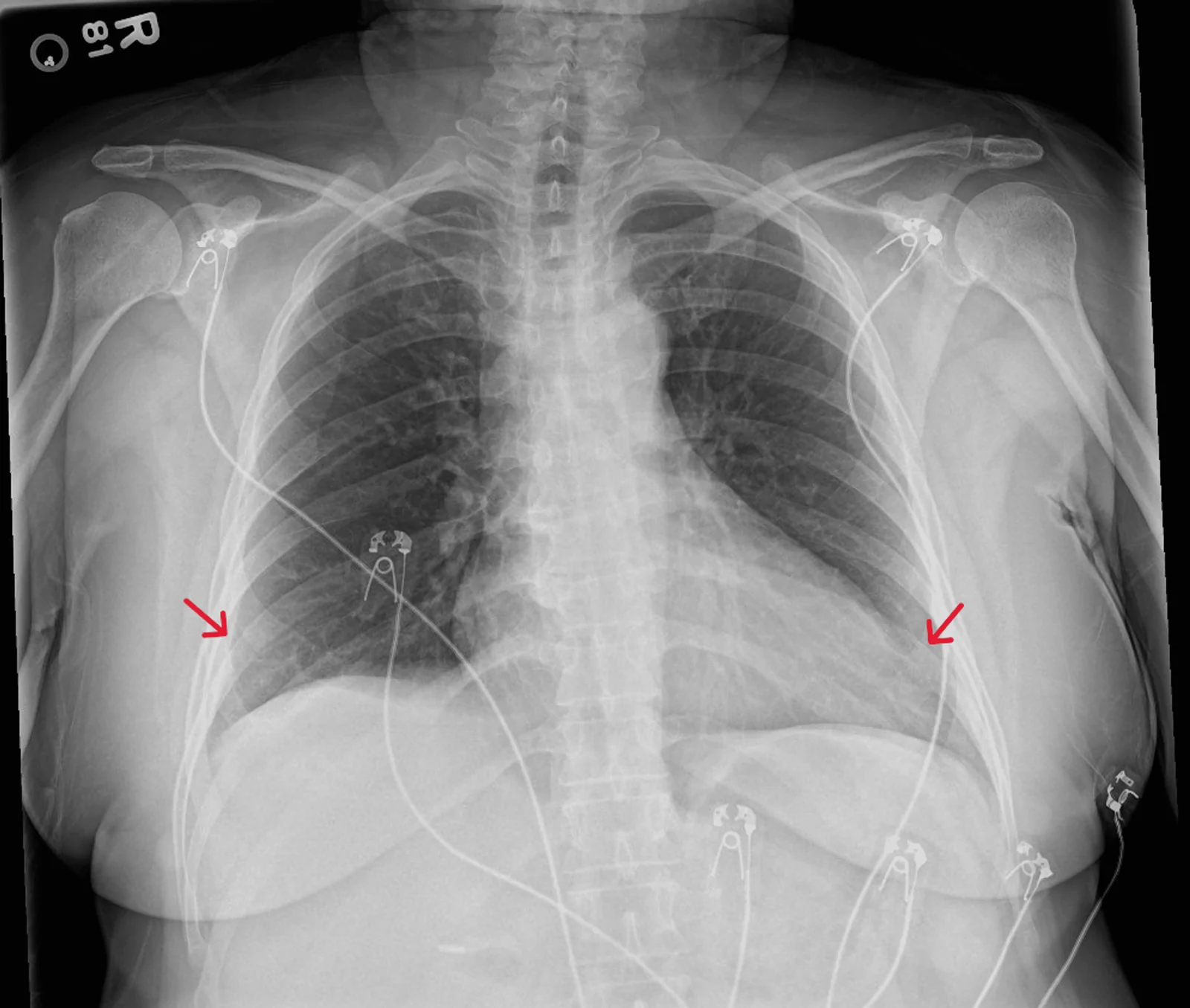
Repeat chest radiograph obtained during the patient's second emergency department presentation, demonstrating no acute cardiopulmonary abnormality. Red arrows indicate the regions of the previously identified bibasilar opacities, which had resolved, with no focal air-space consolidation or other acute cardiopulmonary abnormality.

**Figure 5:**
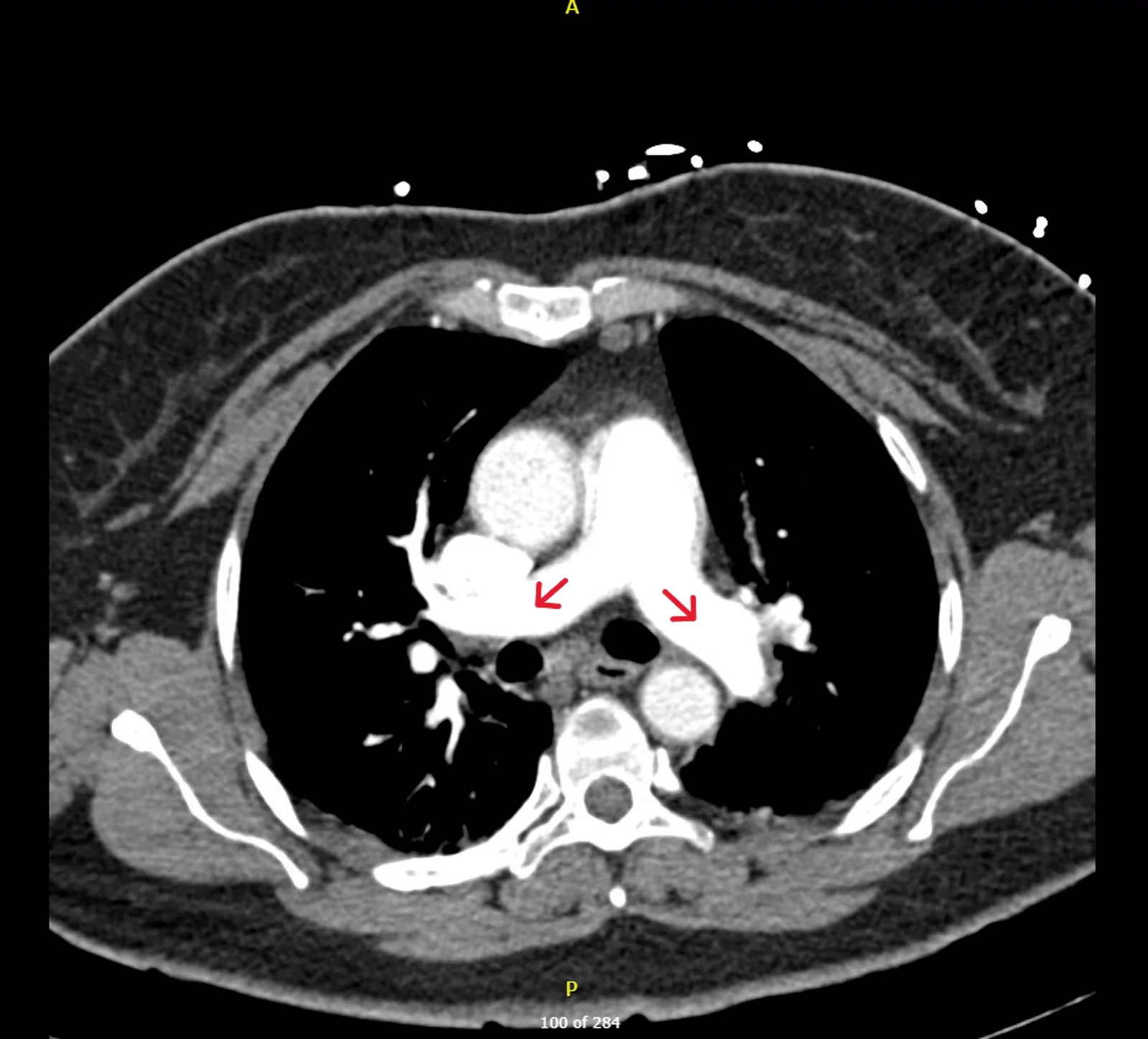
Computed tomography pulmonary angiography demonstrating no evidence of acute pulmonary embolism. Representative axial slice obtained during the patient's second emergency department presentation. Red arrows indicate the contrast-opacified right and left main pulmonary arteries without intraluminal filling defects.

**Figure 6:**
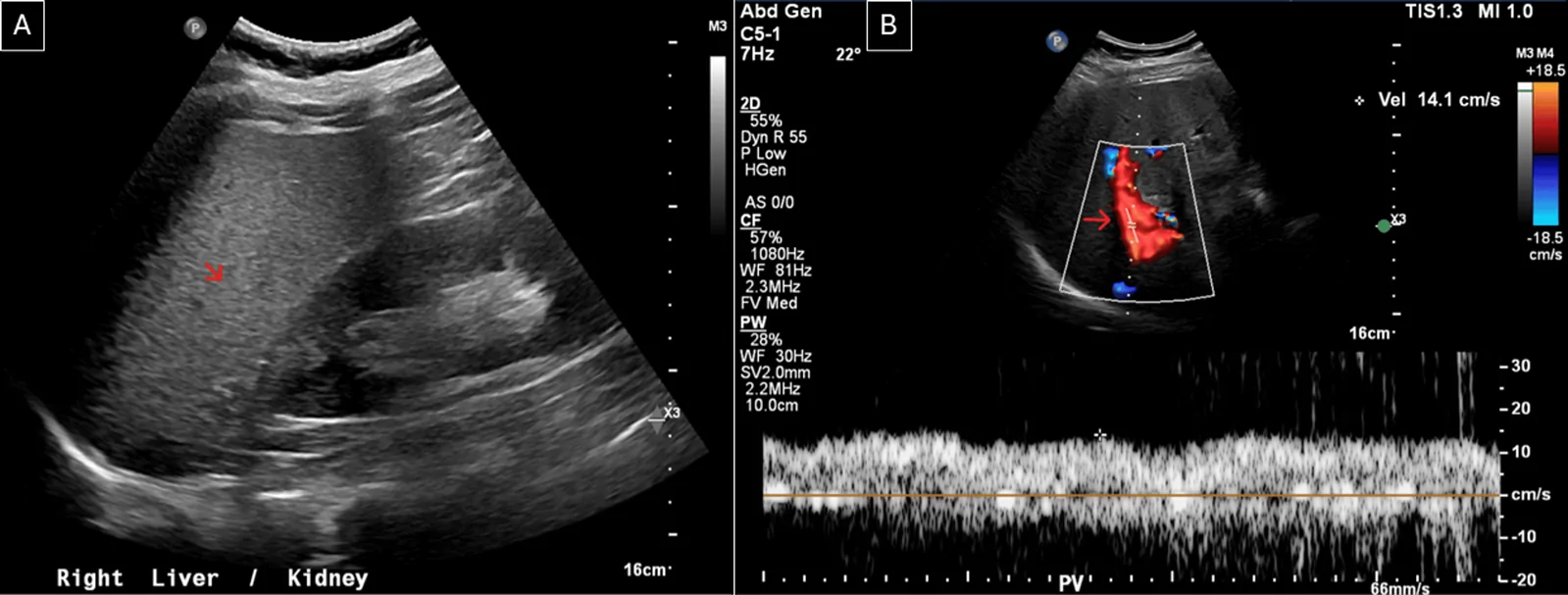
Abdominal ultrasonography demonstrating chronic hepatic steatosis with patent hepatic vasculature. (A) Representative ultrasound image of the right hepatic lobe demonstrating diffusely increased hepatic echogenicity (red arrow), consistent with hepatic steatosis. (B) Color Doppler ultrasound demonstrating a patent main portal vein (red arrow) with preserved hepatopetal flow.

Electrocardiography (Figure 3) demonstrated no acute ischemic changes, and high-sensitivity cardiac troponin I was only mildly elevated, peaking at 33 ng/L before subsequently declining. Repeat chest radiography demonstrated no acute cardiopulmonary abnormality (Figure 4). Computed tomography pulmonary angiography showed no evidence of pulmonary embolism (Figure 5). Transthoracic echocardiography demonstrated normal biventricular systolic function with a left ventricular ejection fraction of 63%. Abdominal ultrasonography performed on the first hospital day demonstrated hepatic steatosis (Figure 6A), previously documented in 2015, with patent hepatic vasculature (Figure 6B).

An extensive evaluation for alternative infectious etiologies, including viral hepatitis, cytomegalovirus, herpes simplex virus, varicella-zoster virus, respiratory viral pathogens, and human immunodeficiency virus, was unrevealing. Blood cultures were negative for bacterial and fungal growth. Given the marked hyperferritinemia and cytopenias, secondary hemophagocytic lymphohistiocytosis (HLH) was considered; however, despite reduced natural killer cell cytotoxic activity, the patient had an HScore of 136, well below the diagnostic cutoff of 169, and lacked additional clinical and laboratory features of HLH. The overall presentation was therefore considered inconsistent with this diagnosis.

Treatment

In light of the close temporal relationship between symptom onset and weekly rifapentine/isoniazid administration, together with the exclusion of alternative infectious, cardiac, hematologic, and autoimmune etiologies, therapy with rifapentine and isoniazid was discontinued on the day of admission. The patient received supportive management consisting of intravenous fluids, close clinical observation, and serial laboratory monitoring. Although suspicion for a concomitant respiratory infection was low, she received a five-day course of empiric doxycycline for possible atypical pneumonia while the diagnostic evaluation was ongoing, with antibiotic selection guided by local antimicrobial resistance patterns.

**Figure 7:**
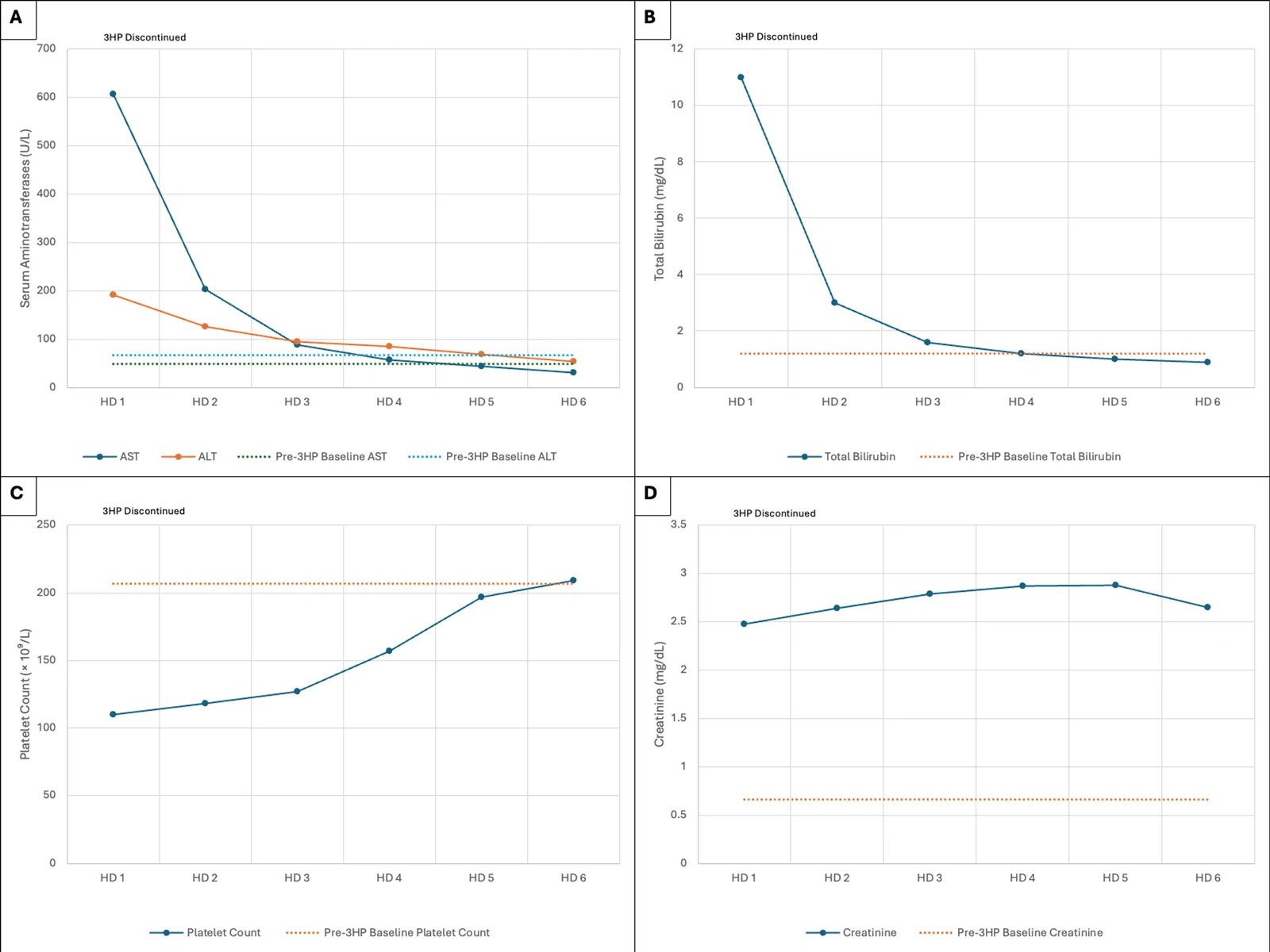
Serial laboratory trends during hospitalization following discontinuation of rifapentine and isoniazid therapy (3HP) (A) Serial serum aminotransferase levels. (B) Serial total bilirubin levels. (C) Serial platelet counts. (D) Serial serum creatinine levels. Dashed horizontal lines represent the patient's pre-treatment baseline values obtained several months before initiation of 3HP regimen. HD: Hospital Day; AST: Aspartate aminotransferase; ALT: Alanine aminotransferase

During hospitalization, hepatic injury and hematologic abnormalities improved steadily with progressive normalization of serum aminotransferases (Figure 7A), total bilirubin (Figure 7B), and platelet count (Figure 7C). Renal recovery was more gradual, with serum creatinine peaking at 2.88 mg/dL before demonstrating improvement towards the end of the hospitalization (Figure 7D). The patient remained non-oliguric and did not require renal replacement therapy. Serial trends in the patient's key laboratory parameters throughout hospitalization are summarized in Table 1.

**Table 1:** Serial laboratory values during hospitalization HD: Hospital day; AST: aspartate aminotransferase; ALT: alanine aminotransferase; LDH: lactate dehydrogenase; BUN: blood urea nitrogen; eGFR: estimated glomerular filtration rate; WBC: white blood cell count.

Laboratory parameter	Reference Range	Pre-3HP Baseline	HD 1	HD 2	HD 3	HD 4	HD 5	HD 6
AST (U/L)	13-62	48	607	203	88	56	43	30
ALT (U/L)	8-70	66	192	126	95	84	68	53
Total Bilirubin (mg/dL)	0.1-1.2	1.2	11.0	3.0	1.6	1.2	1.0	0.9
Conjugated Bilirubin (mg/dL)	≤0.3	not measured	4.3	1.2	0.8	0.6	0.6	not measured
LDH (U/L)	125-256	not measured	2740	1722	983	797	609	not measured
Haptoglobin (mg/dL)	21-210	not measured	<10	<10	<10	30	75	not measured
Hemoglobin (g/dL)	11.6-15.2	14.3	10.1	10.4	9.9	10.5	11.1	9.9
Ferritin (ng/mL)	8-180	not measured	>20000	19747	8645	not measured	1630	not measured
Platelets (10⁹/L)	143-398	207	110	118	127	157	197	209
Creatinine (mg/dL)	0.60-1.30	0.66	2.48	2.64	2.79	2.87	2.88	2.65
BUN (mg/dL)	7-22	14	43	49	52	61	64	61
eGFR (mL/min/1.73m2)	>89	103	22	21	19	19	18	20
WBC (10^3^/µL)	4.16-9.95	5.63	14.26	12.48	8.17	9.55	11.32	12.32
Absolute Eosinophils (10^3^/µL)	0.00-0.50	0.15	0.07	0.09	0.08	0.10	0.16	0.13

By the time of discharge, her symptoms had resolved, physical examination findings had normalized, and she was discharged home in a stable condition with outpatient follow-up with infectious diseases and primary care for continued monitoring and consideration of alternative LTBI therapy.

## Discussion

This case highlights the need to distinguish the self-limited systemic drug reactions commonly associated with the 3HP regimen from the rare but potentially life-threatening manifestations of multiorgan toxicity. Most systemic reactions present with transient flu-like symptoms that occur within several hours of medication administration and resolve spontaneously within 24 hours [5,6]. In contrast, our patient developed progressive hepatic, hematologic, renal, and systemic inflammatory abnormalities requiring hospitalization and discontinuation of therapy.

The 3HP regimen has demonstrated an excellent overall safety profile, with high treatment completion rates and lower hepatotoxicity than longer isoniazid-based regimens [7]. Although systemic drug reactions have been reported, severe events in large clinical trials were primarily limited to hypotension, syncope, and flu-like illness rather than clinically significant end-organ injury [7]. To our knowledge, simultaneous acute liver injury, hemolysis, thrombocytopenia, hyperferritinemia, and acute kidney injury have rarely been reported in association with 3HP therapy, expanding the recognized spectrum of severe adverse reactions.

Although rifapentine, isoniazid, and pyridoxine were discontinued simultaneously, precluding definitive attribution to a single agent, the clinical presentation was most consistent with a rifamycin-associated systemic drug reaction. Application of the Naranjo Adverse Drug Reaction Probability Scale yielded a score of six for rifapentine, indicating a probable adverse drug reaction [8]. This assessment was further supported by the reproducible onset of symptoms several hours after weekly dosing and increased severity following re-exposure, consistent with previously described systemic reactions to rifapentine-containing 3HP regimens. An independent or synergistic contribution from isoniazid cannot be excluded. Isoniazid most commonly causes isolated hepatotoxicity [9]; however, rare manifestations, including hemolytic anemia, thrombocytopenia, acute kidney injury, and drug-induced hypersensitivity syndromes, have also been reported [10,11]. It is therefore possible that concomitant isoniazid therapy contributed to the severity of the patient's multiorgan presentation, although the temporal pattern of symptom recurrence following intermittent dosing more closely resembles a rifamycin-associated systemic drug reaction. In contrast, pyridoxine serves a purely prophylactic role in the 3HP regimen to prevent isoniazid-induced peripheral neuropathy and has a well-established safety profile; its recognized adverse effects are largely limited to dose-dependent neurotoxicity with chronic high-dose exposure, making it an unlikely contributor [12].

Although the patient had chronic hepatic steatosis predating initiation of the 3HP regimen, which may have contributed to her mildly elevated baseline aminotransferases, it does not adequately explain the abrupt onset of severe multiorgan dysfunction, concurrent hematologic and renal abnormalities, or the reproducible temporal relationship between symptom onset and medication administration. Notably, elevated baseline AST has been associated with an increased risk of hepatotoxicity during 3HP therapy [13], suggesting that the patient's pre-existing mild aminotransferase elevation may have increased her susceptibility to the hepatic component of the reaction. The marked hyperferritinemia, cytopenias, and reduced natural killer cell cytotoxic activity raised concern for secondary hemophagocytic lymphohistiocytosis (HLH) [14]. However, the patient did not fulfill the HLH-2004 diagnostic criteria, which require five of eight criteria in the absence of a molecular diagnosis. Although hyperferritinemia and reduced natural killer cell activity were present, she remained afebrile (maximum temperature, 37.2°C), had no splenomegaly, and had a triglyceride level of 188 mg/dL, below the threshold (≥265 mg/dL). Her HScore was also 136, below the validated diagnostic threshold of 169. Furthermore, she improved rapidly following discontinuation of rifapentine and isoniazid with supportive care alone, without corticosteroids or other HLH-directed immunosuppressive therapy, further arguing against HLH and supporting a severe drug-induced hypersensitivity reaction.

The significance of the patient's transient bibasilar pulmonary opacities remains uncertain. Although a concurrent community-acquired pneumonia could not be definitively excluded, the reproducible onset of symptoms shortly after medication administration, spontaneous resolution despite not taking the initially prescribed antibiotics, negative subsequent infectious evaluation, and overall clinical course raise the possibility that these pulmonary findings represented part of the systemic inflammatory response rather than a primary pulmonary infection.

The mechanism underlying severe systemic reactions to rifapentine/isoniazid therapy remains incompletely understood but is thought to involve immune-mediated hypersensitivity, cytokine-mediated inflammation, and drug-dependent antibody formation [15,6]. Intermittent drug exposure may promote sensitization, explaining why symptoms typically occur after subsequent doses rather than after the initial exposure [16]. Notably, hepatic and hematologic abnormalities improved rapidly following drug discontinuation, whereas renal recovery was delayed, suggesting that different organ systems may recover at different rates despite removal of the offending agents [17-19].

This case emphasizes several important clinical implications. Clinicians should suspect a medication-related reaction in patients receiving the 3HP regimen who develop recurrent systemic symptoms shortly after dosing, particularly when symptoms worsen with repeated exposure. Early recognition, prompt discontinuation of therapy, and supportive management are critical to prevent progression to severe multiorgan injury and improve patient outcomes.

## Conclusions

Although the 3HP regimen is generally safe and well tolerated, clinicians should be aware that rare cases of severe multiorgan toxicity can occur. This case highlights a probable drug-related reaction in which recurrent systemic symptoms shortly after medication administration heralded significant hepatic, hematologic, renal, and inflammatory involvement. Early recognition of a potential drug-related reaction, prompt discontinuation of therapy, and supportive management are essential to prevent further organ injury and improve patient outcomes.
